# Thriving Under Stress: *Pseudomonas aeruginosa* Outcompetes the Background Polymicrobial Community Under Treatment Conditions in a Novel Chronic Wound Model

**DOI:** 10.3389/fcimb.2020.569685

**Published:** 2020-10-06

**Authors:** Joann Phan, Saba Ranjbar, Miki Kagawa, Matthew Gargus, Allon Israel Hochbaum, Katrine L. Whiteson

**Affiliations:** ^1^Department of Molecular Biology and Biochemistry, University of California, Irvine, Irvine, CA, United States; ^2^Department of Chemical and Biomolecular Engineering, University of California, Irvine, Irvine, CA, United States; ^3^Department of Materials Science and Engineering, University of California, Irvine, Irvine, CA, United States; ^4^Department of Chemistry, University of California, Irvine, Irvine, CA, United States

**Keywords:** polymicrobial, wound model, *Pseudomonas aeruginosa*, volatile organic compound (VOC), antibiotics, hydrogen peroxide

## Abstract

*In vitro* infection models are important for studying the effects of antimicrobials on microbial growth and metabolism. However, many models lack important biological components that resemble the polymicrobial nature of chronic wounds or infections. In this study, we developed a perfused meat model that supports the growth of the human pathogen *Pseudomonas aeruginosa* in a native meat microbial background to investigate the impact of antibiotics and hydrogen peroxide on polymicrobial community growth and metabolism. *P. aeruginosa* plays an important role as an etiological agent involved in chronic infections and is a common opportunistic pathogen. Chemical stressors in the form of hydrogen peroxide, carbenicillin, and gentamicin were perfused through the meat with polymicrobial growth on the surface. The relative abundances of *P. aeruginosa* and the background microbial community were analyzed by cell viability assays, and metabolic changes of the entire community in response to different antimicrobial treatments were characterized by GC-MS analysis of volatile organic compounds. The meat background community was characterized by amplicon sequencing. Relative densities of *P. aeruginosa* and background microbiota were similar under control conditions. Antimicrobial stressors, even at sub-inhibitory, physiologically relevant concentrations, spurred *P. aeruginosa* dominance of the meat surface community. Volatile metabolite ion intensity levels showed that antibacterial treatments drive changes in microbial metabolism. The abundance of the *P. aeruginosa-*derived metabolite, acetophenone, remained stable with treatment, whereas the relative abundances of 2-butanone, 2-nonanone, and 2-aminoacetophenone changed in response to treatment, suggesting these could serve as biomarkers of infection. Our model recapitulates some of the physiological conditions of chronic wounds and facilitates high throughput experiments without the high cost of *in vivo* models. Expanded use of this perfusion model will contribute to the understanding of polymicrobial growth and metabolism in the context of chronic wounds and infections.

## Introduction

Chronic wounds, characterized by the impairment or disruption of the natural wound healing process, are problematic to identify and treat because of the complex underlying physiology of each individual. A majority of chronic wounds are infected with bacteria that are resistant to antimicrobial challenge (Cochran et al., 2000; Hernandez, 2006). Chronic wound infections are more persistent than acute infections due to host pathophysiologies and bacterial tolerance to physical and chemical therapies, including increased resistance to antibiotics. Although the wound microbiome has been well-characterized (Dowd et al., 2008a,b; Thomsen et al., 2010; Wolcott et al., 2016), defining the composition of the chronic wound microbiome may not enable robust prediction of wound outcome or healing. However, multispecies communities in an *in vivo* wound model have been shown to delay healing compared to single species infections (Dalton et al., 2011). Metabolic biomarkers that result from microbial interactions may provide important insight into chronic wound metabolism and progression. Several *in vitro* and *ex vivo* studies have documented the potential clinical relevance and reliability of volatile molecules in distinguishing microbes based on their volatile signatures and exploring the microbial metabolisms of chronic wounds (Byun et al., 2010; Thomas et al., 2010; Jia et al., 2014; Ashrafi et al., 2017, 2018). The use of GC-MS to identify volatile organic compounds (VOC) can provide important insight into microbial metabolism and physiology. Thus, in order to improve chronic wound diagnosis and treatment, it is essential to develop realistic and high throughput models to better understand the physiology and metabolic signatures of chronic infection.

Opportunistic pathogens such as *Pseudomonas aeruginosa* typically dominate the microbial community in chronic infections, yet the underlying mechanisms are not well-understood. In the earlier stages of infection, commensal microbes and host physiology play an important role in establishing a favorable environment for the opportunistic pathogen. For example, in cystic fibrosis, anaerobes present in the airways break down mucins to produce amino acids and short chain fatty acids that *P. aeruginosa* uses as a carbon source to grow and colonize (Flynn et al., 2016). Cross-feeding between commensal bacteria and *P. aeruginosa* has also resulted in changes in antibiotic tolerance (Adamowicz et al., 2018). However, the dynamic between the commensal microbes and opportunistic pathogens is missing in many chronic wound and infection models. Several wound studies indicate multispecies biofilms have negative impacts on wound healing and antimicrobial efficacy (Dalton et al., 2011; Oates et al., 2018). In an *in vivo* murine chronic wound model, oxidative stress and community composition alone do not characterize the microbial community metabolism (Dhall et al., 2014). Similarly, tissue models, including an *ex vivo* porcine lung model, investigate only the growth of a single pathogen at a time (Harrison et al., 2014; Dumigan et al., 2019; Harrington et al., 2020; Sweeney et al., 2019). To better understand chronic infections, it is important to consider the entire microbial community.

**Figure 1:**
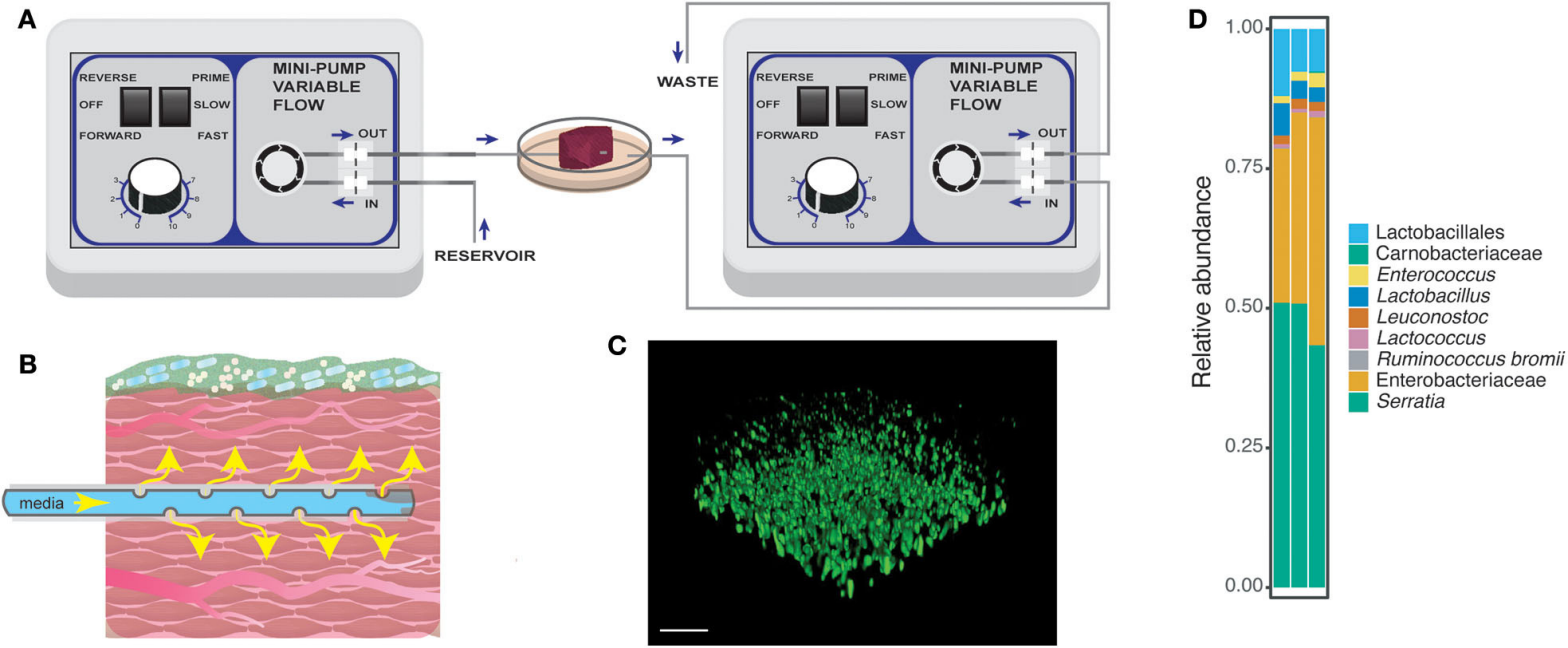
Schematic for the perfusion wound meat model. **(A)** Perfusion setup with two flow regulators as described in the section **Methods**. Briefly, the purpose of each of the flow regulators was to introduce flow of nutrients through the tissue and to remove excess media waste. Perforated tubing was threaded through the tissue to provide nutrients to the surrounding tissue. The end of the tube was capped, allowing the media to be released only through the pores on the tubing in the meat [also displayed in **(B)**]. **(B)** A closer look at the tissue with flow and microbial community growth from the perfusion model. **(C)** A confocal image of the PA14-*yfp* growing on the surface of the tissue. The image was taken from the static model after 24 h of growth. Bar = 20 μm. The tissue had a native microbial community, meat background (MB control), whose bacterial members are displayed in **(D)**. **(D)** Amplicon sequencing was performed on MB control communities harvested from three replicates from the static model.

In order to model a polymicrobial community in a biologically relevant context, we developed a perfusion wound meat model that could support the growth of a native meat microbial background and *P. aeruginosa* (Figure 1). Based on an *ex vivo* porcine lung model for cystic fibrosis (Harrison et al., 2014; Sweeney et al., 2019), our *in vitro* model adds liquid flow via media perfusion and a native meat microbial community. Dumigan et al. (2019) included perfusion in the *ex vivo* porcine lung model, but investigated the pathogenesis of a single pathogen – *Klebsiella pneumoniae* without the presence of a native microbiota. Oates et al. (2018) developed a basally perfused model that successfully supports the growth of a multispecies community, but lacks a biological host component. Our perfused meat model incorporates nutrient flow through a meat matrix that supports the growth of a multispecies community. Just as human skin harbors a native microbial community, the meat microbial community was used to replicate a non-sterile environment of a wound or chronic infection.

To study changes in metabolite profiles in these microbial communities, volatile molecules were detected using vacuum assisted sorbent extraction (VASE). VASE involves extracting volatile molecules under vacuum, which results in greater detection sensitivity than methods that actively absorb the volatiles from the headspace of a sample (i.e., solid phase microextraction). The measurement of volatile molecules provides a non-invasive approach that could be widely used for detecting biomarkers in health and disease. Other studies have incorporated surveying volatile molecules to identify biomarkers of chronicity in wounds (Byun et al., 2010; Thomsen et al., 2010; Ashrafi et al., 2018). Therefore, elucidating the metabolism within the polymicrobial communities in wounds in response to antibiotic treatment and ROS from the host may reveal important information on treatment efficacy, microbial persistence, and host outcome.

We used the perfusion meat model to address the following questions: (1) how does the microbial community composition and viability shift in response to common wound therapies (antibiotics and hydrogen peroxide), and (2) how do metabolic signatures of this community change in response to these therapies? The overall technological goal and impact was to understand the community, metabolite, and pathogen dynamics in bacterial infections.

## Materials and Methods

### Strains and Growth Conditions

*Pseudomonas aeruginosa* PA14 WT (Rahme et al., 1995) and clinical isolate PaFLR01 were used in this study. PA14 constitutively expressing YFP (provided by R. Kolter, Harvard Medical School) was used to visualize growth on the surface of the meat tissue. Clinical isolate PaFLR01 was isolated from the sputum of an individual with cystic fibrosis. The genome sequencing and assembly can be found with BioProject Accession PRJNA434465 on NCBI. Published studies involving PaFLR01 include Phelan et al., 2015; Phan et al., 2017, and Phan et al., 2018. *P. aeruginosa* strains were inoculated into Luria-Broth (LB for PA14 and PA14-*yfp*; Sigma-Aldrich) or Todd-Hewitt broth (TH broth for PaFLR01; Sigma-Aldrich) and grown overnight on a shaker at 37°C. PaFLR01 does not grow well on LB, thus requiring a richer media for growth. Optical density measurements to estimate bacterial culture concentrations were made at 500 nm, rather than the standard 600 nm, in order to avoid overlapping signals from pigments including pyocyanin produced by *P. aeruginosa*. The meat microbial background was native to the meat.

### Perfusion Meat Model

Meat for the perfusion model was from a packaged fresh (never frozen) New York Strip Steak from a commercial source (beef, Trader Joe's). Steak tissue was cut into ~1 inch cubes and stored in phosphate buffered saline (PBS) at −20°C and thawed at 4°C before use. At the start of the experiment, the tissue was submerged in *P. aeruginosa* from an overnight culture diluted in LB to OD_500nm_ = 0.05, or sterile LB as a control, in a 50 mL tube for 2 h at 37°C before setting up the perfusion model. At the end of this incubation, the tissue was transferred to a 30 mm petri dish for the perfusion setup.

The setup consisted of tubing, two flow regulators, a 30 mm petri dish, an autoclave tub, tissue, and 500 mL glass bottles to store media or collect waste (Figure 1A, Supplementary Figure 1). To pass the tubing through the 30 mm petri dish, a heated 16 ½ G needle was used to make holes on opposite sides of the dish. We pipetted 4 ml of LB into the 30 mm petri dish, threaded tubing that was perforated and sealed at one end through one side of the petri dish and tissue. The tubing that collected waste or excess media was pulled through the opposite side of the petri dish and the end of the tube was placed where the outward flow would not be disrupted. The threading process was performed with autoclaved dissection tweezers. Liquid media continuously flowed through the tube at the rate of 0.2 ml/min. Waste collection was also set at the rate of 0.2 ml/min. The perfusion setup was incubated for 24 h in a temperature controlled room set at 37°C.

Perturbation conditions included the following additions to the input media: 50 μg/ml carbenicillin, 20 μg/ml gentamicin, or various concentrations of hydrogen peroxide. The concentrations of gentamicin and hydrogen peroxide were selected based on concentrations found in the literature relevant to chronic wounds and wound treatments summarized in Supplementary Table 1. At the conclusion of each perfusion experiment, half of the tissue was saved for enumeration of colony forming units (CFUs) and the other half was saved for analysis of volatile compounds.

Sterilization of the materials used in the perfusion model involved the use of bleach, ethanol, and autoclaving. The tubing was submerged in a 10% bleach solution for at least 2 h, rinsed and drained with water, dried, and autoclaved. The surgical tweezers used for manipulation of the tubing and meat were sprayed with bleach and autoclaved prior to each experiment. During the perfusion setup, the tweezers were sprayed with a bleach solution and then an ethanol solution to prevent cross-contamination when preparing multiple experiments. The petri dishes were purchased as sterile.

### Static Meat Model

The basis of this experimental setup was modeled after the *ex vivo* porcine lung model in Harrison, F. et al. 2014 (Harrison et al., 2014; Dumigan et al., 2019; Sweeney et al., 2019). The differences, however, included the tissue source and media, and our model did not use antibiotics to clear the native microbial community from the tissue. Tissue for the static model was the same as used for the perfusion model. The static model was performed in a 12-well plate and stored at 37°C for 24 h. The tissue was submerged in a diluted overnight bacterial culture (OD_500nm_ 0.05), or sterile LB as a control, in a 50 mL tube and incubated for 2 h prior to the start of the plate experiment. After the 2 h incubation, each cube was placed in an individual well in the 12-well plate. Media was added to each well to cover the tissue. Each well contained about 2 ml media with or without antibiotic or hydrogen peroxide and the tissue. At the conclusion of each static experiment, half of the tissue was saved for enumeration of CFUs.

### Cell Viability and Relative Growth Analysis

Half of the tissue was vortexed with 1 ml of media for 30 s to remove cells adhered to the tissue surface. The liquid was then collected and used for dilution plating, where 10 μl of each dilution was spotted onto a LB or TH agar petri dish at least three times as technical replicates for each biological sample. LB agar plates were used for models with PA14, whereas TH agar plates were used for models with PaFLR01. Colonies of *P. aeruginosa* and meat background (MB) microbes were distinguished by phenotype. *P. aeruginosa* formed smaller and darker brown-green colonies while MB formed larger white colonies. All colonies that were not the *P. aeruginosa* phenotype were counted as MB.

### Statistical Analyses

All statistical analyses were performed in R (R Core Team, 2017). Analysis of variance (ANOVA) was performed with the aov function. Corrections for multiple comparisons following ANOVA were performed with the TukeyHSD function. Assumptions for ANOVA were tested with shapiro.test (normal distribution of residuals) and leveneTest (car package; homogeneity of variances) (Fox et al., 2020). For data that did not meet both assumptions for ANOVA, *T*-tests were performed. The compare_means function from the ggpubr package in R was used to perform *T*-tests to compare group means (Alboukadel, 2018). *P*-values were adjusted with the Benjamini-Hochberg method (*p*.adjust.method = “BH”) for tests with multiple comparisons. Non-metric multidimensional scaling (NMDS) was performed with the metaMDS function from the vegan package in R (Oksanen et al., 2018). To generate clusters in the metabolic heatmap, a Bray-Curtis distance matrix of the sample normalized metabolite data was generated using the vegdist function in the vegan package (Oksanen et al., 2018). The resulting matrix was then put into dist, hclust, and as.dendrogram functions from the package stats (R Core Team, 2017).

### Headspace Detection and Analysis

Volatile headspace analysis was performed using vacuum assisted sorbent extraction (VASE); the instrument and method developed by Entech Instruments (Simi Valley, California) were coupled with sample injection and thermal desorption on a gas chromatography mass spectrometer (GC-MS). The half of the tissue sections not used for viability and growth analysis were prepared by VASE for 1 h at 70°C. Briefly, tissues stored at −80°C were thawed on ice for 1 h and then placed into a pre-cleaned type 1 glass vial (VOA; Thermo Scientific). A VASE Sorbent Pen cartridge containing Tenax was placed into the vial and held in place by a lid liner. Air was removed from the vials using a vacuum pump and the vials were placed in a shaking incubator for 1 h at 70°C. At the end of the extraction, vials were placed on a metal block equilibrated at −20°C for 15 min to remove water from the headspace. VASE pens were removed from the vials and their contents were run on an Agilent GC-MS (7890A GC and 5975C inert XL MSD with Triple-Axis Detector) with a DB-624 column. The splitless GC-MS method starts at 35°C with a 5 min hold, ramps 10°C/min until 170°C, and ramps 15°C/min until 230°C with a total method runtime of 38 min. The corresponding Entech method has a 38 min runtime with a pre-heat duration for 2 min at 260°C, desorption duration for 2 min at 260°C, bake-out duration for 33 min at 260°C, and post-bake duration for 3 min at 70°C. Analysis and quantification of peaks were performed in the Agilent ChemStation software.

### Meat Microbial Background Characterization

The meat microbial background was harvested from the control static meat model after 24 h at 37°C. Microbial cells were collected from two sources: (1) the media the tissue was incubated in and (2) cells attached to the tissue were collected by washing and vortexing the tissue with fresh media. Communities from three pieces of meat from the same steak were sequenced. All materials collected were combined, pelleted, and stored at −80°C until DNA extraction. DNA was extracted using the Quick-DNA Fecal/Soil Microbe Miniprep kit (D6010) from Zymo Research (Irvine, CA). The 16S rRNA V4–V5 region was amplified using the 515F and 926R primers from the Earth Microbiome Project (Walters et al., 2015) and sequenced on an Illumina Miseq. The composition was determined using QIIME2 (*https://qiime2.org/*). The 16S rRNA data are available on the SRA at NCBI with BioProject ID PRJNA640161.

### Visualization of PA14 Growth on Meat Tissue Surface

To visualize growth on the surface of the meat, we grew PA14-*yfp* in the static model for 24 h. The tissue was rinsed gently with 1X PBS to wash off excess cells from the surface. Confocal laser scanning microscopy (Zeiss LSM780) with 63X oil immersion was used to visualize PA14-*yfp* growth on the surface of the tissue. Dichroic beam splitters were used to filter laser lines at 488 nm and emission intensity was collected from YFP at 493–598 nm. The images were processed in Volocity software to show the 3D image of PA14-*yfp* growth on the tissue.

## Results

### Development of a Novel Perfused Meat Model

The goal of the perfused meat model was to create a three-dimensional *in vitro* model of bacterial community growth on an organic substrate resembling the wound environment to study the effects of antimicrobials on microbial growth and metabolism (Figures 1A,B). Perfusing the tissue with nutrients mimics the role of vasculature found in wound tissue; the flow rate of media can be tailored to mimic different tissue types and could be infused with different compounds of interest such as host-derived factors and therapeutics, including the antibiotics and reactive oxygen species used in the present study. Pictures of the perfusion setup are shown in Supplementary Figure 1. Visualization of PA14-*yfp* on the surface of the meat indicates that this strain is able to grow on and adhere to the surface of the meat (Figure 1C). The use of steak as tissue provides important biological and structural or physical components that would not be present in a liquid culture model. Steak also has a native polymicrobial community, which was dominated in our samples by Enterobacteriaceae and *Serratia* (Figure 1D) but also included Lactobacillales, Carnobacteriaceae, *Enterococcus, Lactobacillus, Leuconostoc, Lactococcus*, and *Ruminococcus bromii*. In the following, we refer to this community as the meat background (MB). The microbes characterized in the meat background are also found as normal flora of the human gut, skin, oral cavity, and vagina, suggesting that the microbes native to the perfused meat model may represent relevant interactions for *P. aeruginosa* in infections in different types of communities.

In the meat model experiments described below, we grew and analyzed communities composed of either the MB alone or the MB inoculated with one of two *P. aeruginosa* strains. We refer to each component using the following nomenclature: MB alone = “MB control,” community with PA14 and meat background = “PA14+MB,” community with PaFLR01, and meat background = “PaFLR01+MB,” PA14 in the presence of meat background = “PA14(MB),” PaFLR01 in the presence of meat background = “PaFLR01(MB),” meat background in the presence of PA14 = “MB(PA14),” and meat background in the presence of PaFLR01 = “MB(PaFLR01).” We included clinical isolate PaFLR01 in order to explore whether the relative competition with the MB could be extended to a clinically relevant isolate in the context of treatment stress.

### Impact of Antimicrobial Compounds on the Growth of the *P. aeruginosa* and Meat Microbial Background

**Figure 2:**
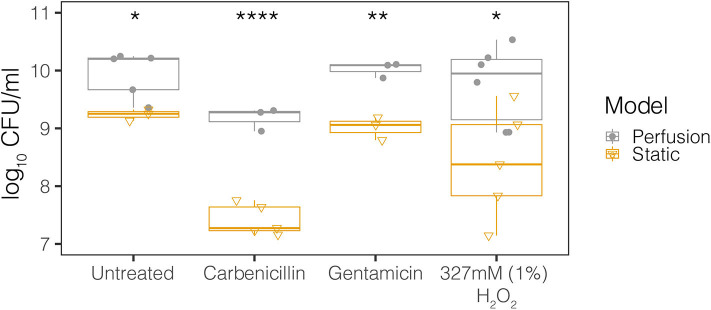
Perfusion and static model growth comparisons for the MB control community across antimicrobial treatments. The MB control community is the microbial background native to the meat. Treatments include control, 327 mM (1%) hydrogen peroxide, 50 μg/ml carbenicillin, and 20 μg/ml gentamicin. The closed circles represent the perfusion model and the open triangles represent the static model. Each point represents the average from at least three technical replicates from dilution plating for each biological replicate (*N* ≥ 3). Statistical testing was performed with ANOVA. Significance is indicated by asterisks. *p < 0.05; **p < 0.01; ****p < 0.0001.

In order to rationalize the use of media perfusion in our model, we first compared perfusion and static conditions for the MB control population in our meat model. The concentrations of antibiotics and hydrogen peroxide (mM range) were chosen based on those used in therapeutic treatment and, in the case of hydrogen peroxide, produced under physiological conditions (μM range) (Supplementary Table 1). The MB control population had significantly higher viable cell counts when grown in perfused media conditions compared to static conditions for all treatments – control, 327 mM (1%) hydrogen peroxide, carbenicillin, and gentamicin (Figure 2). All subsequent experiments were performed under perfused media conditions.

**Figure 3:**
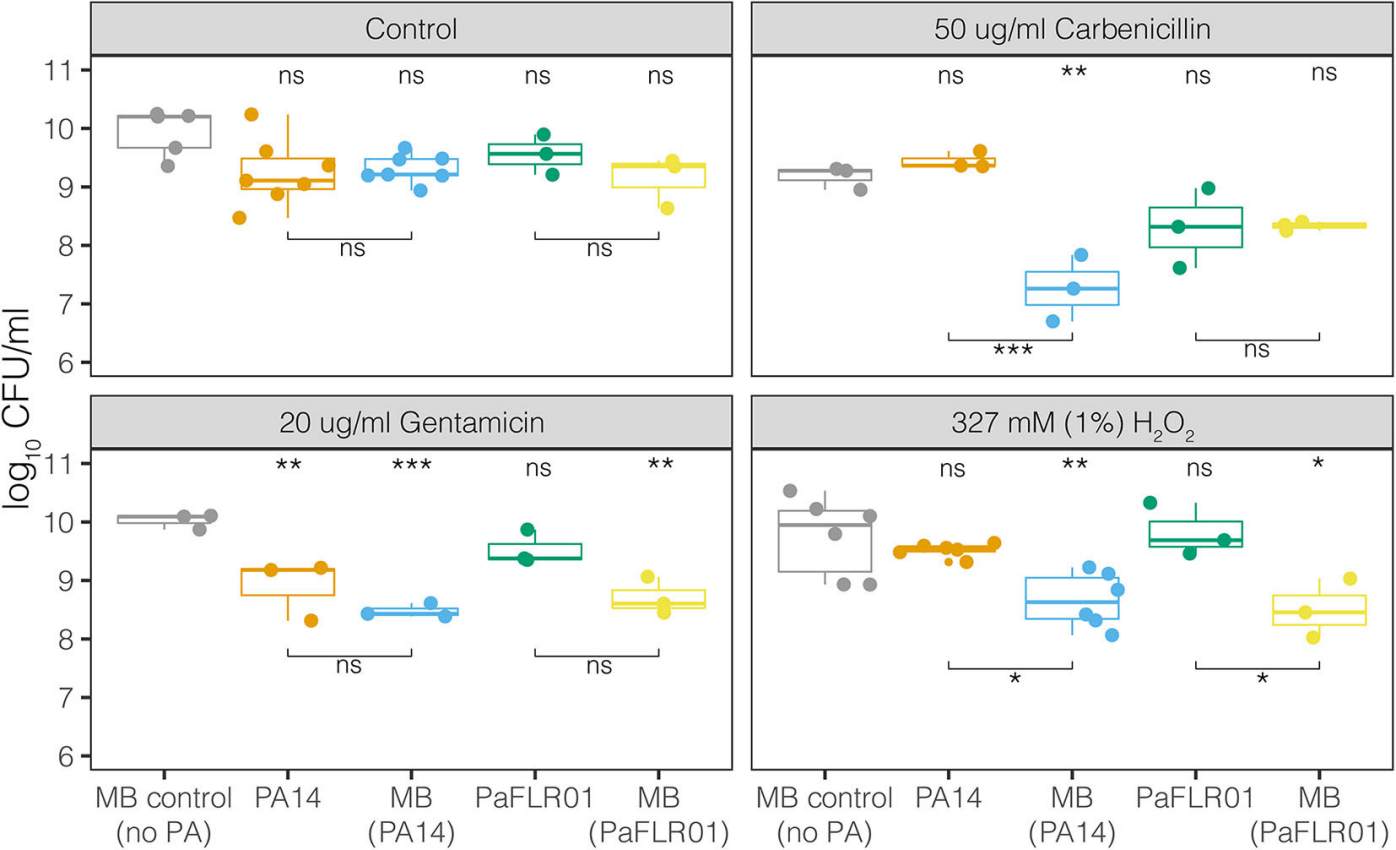
Growth, in CFU/mL, of MB alone, *P. aeruginosa* strains [PA14, PaFLR01] and their corresponding MB communities [MB(PA14), MB(PaFLR01)] in the perfused media meat model. Each point is a biological replicate and represents the average of >3 technical replicates from dilution plating. *N* ≥ 3 biological replicates for all conditions. Statistical testing was performed with ANOVA with *post-hoc* Tukey tests. Significance is indicated by asterisks. The asterisk and “ns” indicators on the top of the graph are significance tests of each population vs. the MB control (no PA). The asterisks and “ns” indicators with bars below the data are comparisons between the populations connected by the bars. ns *p* > 0.05; *p < 0.05; **p < 0.01; ***p < 0.001.

To measure the effects of antimicrobials on polymicrobial communities in the meat model, we perfused carbenicillin, gentamicin, gentamicin plus hydrogen peroxide, and hydrogen peroxide alone through meat with MB only or PA14+MB or PaFLR01+MB communities and measured colony forming units per ml (CFU/ml) after 24 h of incubation (see section Materials and Methods). In the control conditions, without any antimicrobial treatment, the viable cell density (CFU/ml) did not significantly differ between either *P. aeruginosa* strain and their corresponding meat background community, indicating that the growth of one population did not inhibit the growth of the other (Figure 3). There were also no differences in growth between the MB control (no *P. aeruginosa*) and the *P. aeruginosa* associated MB populations (Figure 3). However, with the addition of antibiotics or hydrogen peroxide at all therapeutic concentrations, there was a significant decrease in the *P. aeruginosa*-associated MBs [MB(PA14) and MB(PaFLR01)], with the exception of the MB(PaFLR01) in carbenicillin (Figure 3 and hydrogen peroxide below 1% in Supplementary Figure 2). On the other hand, the *P. aeruginosa* strains were not inhibited relative to the MB control in all above conditions, with the exception of PA14 in gentamicin (Figure 3).

For additional therapeutically relevant treatments – combinations of both gentamicin and hydrogen peroxide (1%), there was a significant decrease in the MB(PA14) population compared to PA14 and MB control (Supplementary Figure 2) despite there being no significant difference for PA14 and MB(PA14) with gentamicin exposure alone. At H_2_O_2_ concentrations relevant to those naturally occurring in wounds during healing (0.05–0.5 mM) (Roy et al., 2006; Ojha et al., 2008; Loo et al., 2012), there were no significant differences between PA14 and MB(PA14) populations (Supplementary Figure 2). Significant differences between PA14 and MB(PA14) populations occurred at 1mM, 10 mM, 327 mM (1%), and 980 mM (3%) hydrogen peroxide (Supplementary Figure 2).

### Polymicrobial Community Volatile Metabolism

To probe the metabolism of the polymicrobial community when exposed to antimicrobials, we detected volatile metabolites with vacuum assisted sorbent extraction (VASE) and quantified relative abundances of the analytes on a gas chromatography-mass spectrometer (GC-MS). Because PA14 and MB(PA14) were grown on the same tissue, we could not determine whether PA14 or one of the native bacteria produced each volatile metabolite in these samples. However, we did measure the volatile molecules emitted from the MB control community as a baseline for identifying changes in metabolism associated with the addition of PA14 and antimicrobials. In addition, we expect the volatile molecules associated with fermentation such as acetic acid, 2,3-butanedione, ethanol, acetaldehyde and others, to be more commonly associated with the meat microbial background than PA14, which does not ferment unless in the absence of alternative anaerobic respiratory nutrients such as nitrate, nitrite, or arginine (Vander Wauven et al., 1984; Davies et al., 1989; Eschbach et al., 2004). We also expected to detect core volatiles known to be produced by *P. aeruginosa*, including 2-nonanone, acetophenone, 2-aminoacetophenone, 2-butanone, dimethyl sulfide, dimethyl trisulfide, 2-heptanone, and others (Labows et al., 1980; Shestivska et al., 2012; Bean et al., 2016).

**Figure 4:**
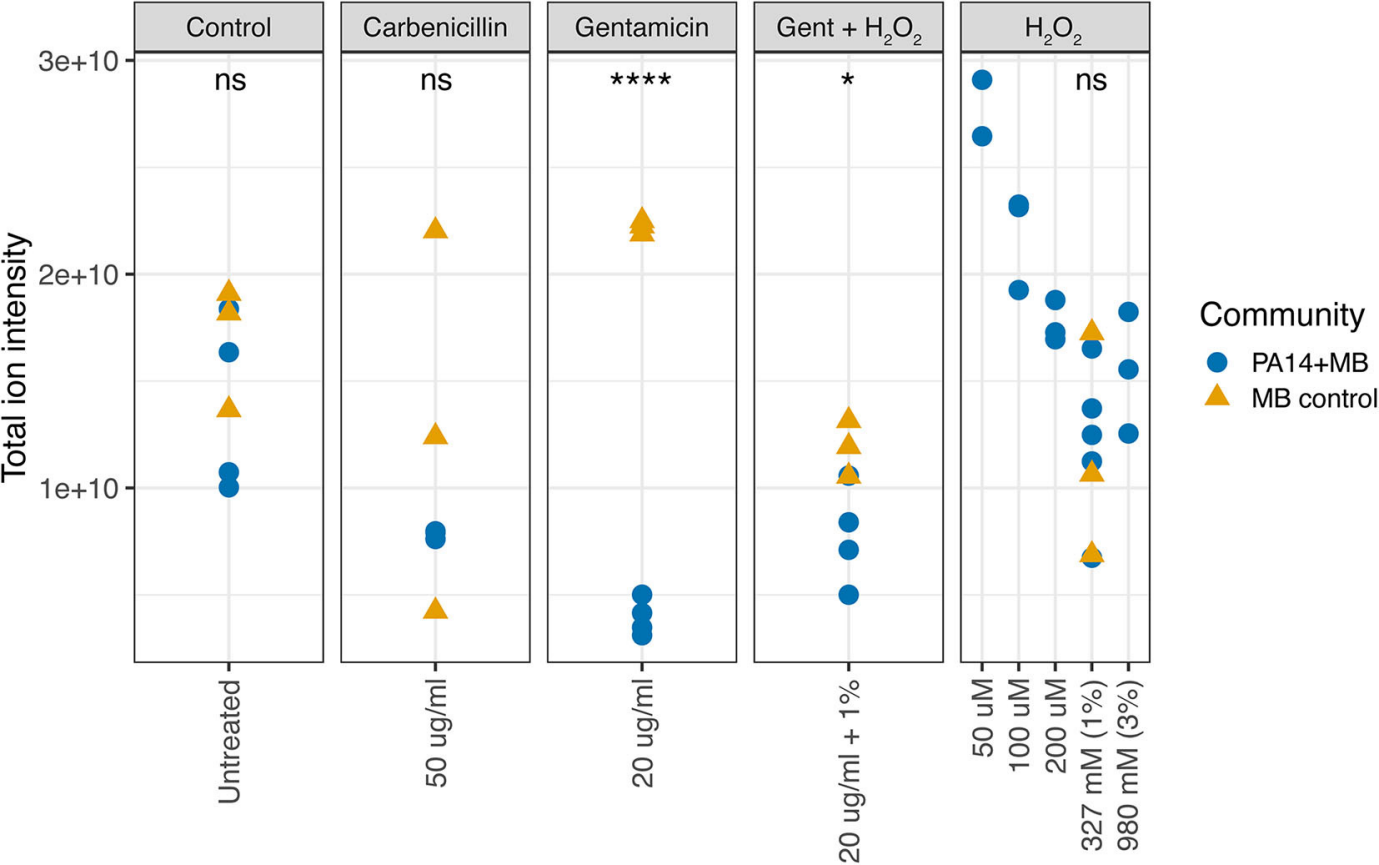
Total ion intensity of all volatile compounds. The PA14+MB community represents the microbial community on a meat sample with both PA14 and MB(PA14) populations. The MB control community is a meat sample with only the meat microbial background. Significant differences were found for gentamicin (*p* = 2.2 × 10^−6^, *T*-test) and gentamicin + H_2_O_2_ (*p* = 0.034, *T*-test).

We first used the VASE data to calculate the overall metabolite intensity for each treatment condition. In the control, carbenicillin, and hydrogen peroxide conditions, there were no significant differences in total ion intensity between MB control and PA14+MB communities (Figure 4). However, the total ion intensity of MB control was significantly higher than PA14+MB when exposed to gentamicin (*p* = 2.2 × 10^−6^) or gentamicin plus hydrogen peroxide (*p* = 0.034) (Figure 4). Interestingly, MB control growth was significantly higher than MB(PA14) with gentamicin and gentamicin plus hydrogen peroxide (Figure 3, Supplementary Figure 2). Because more of the volatile signal may be coming from the MB control community, there may be changes in MB control growth associated with total ion intensity. For the PA14+MB community, low concentrations of hydrogen peroxide (50–100 μM) had the highest total ion intensity. As hydrogen peroxide concentrations increased, total ion intensity decreased (Figure 4). There was a negative correlation between the μM concentrations of hydrogen peroxide and total ion intensity for the PA14+MB communities (Pearson's correlation *R* = −0.9, *p* = 0.0024).

**Figure 5:**
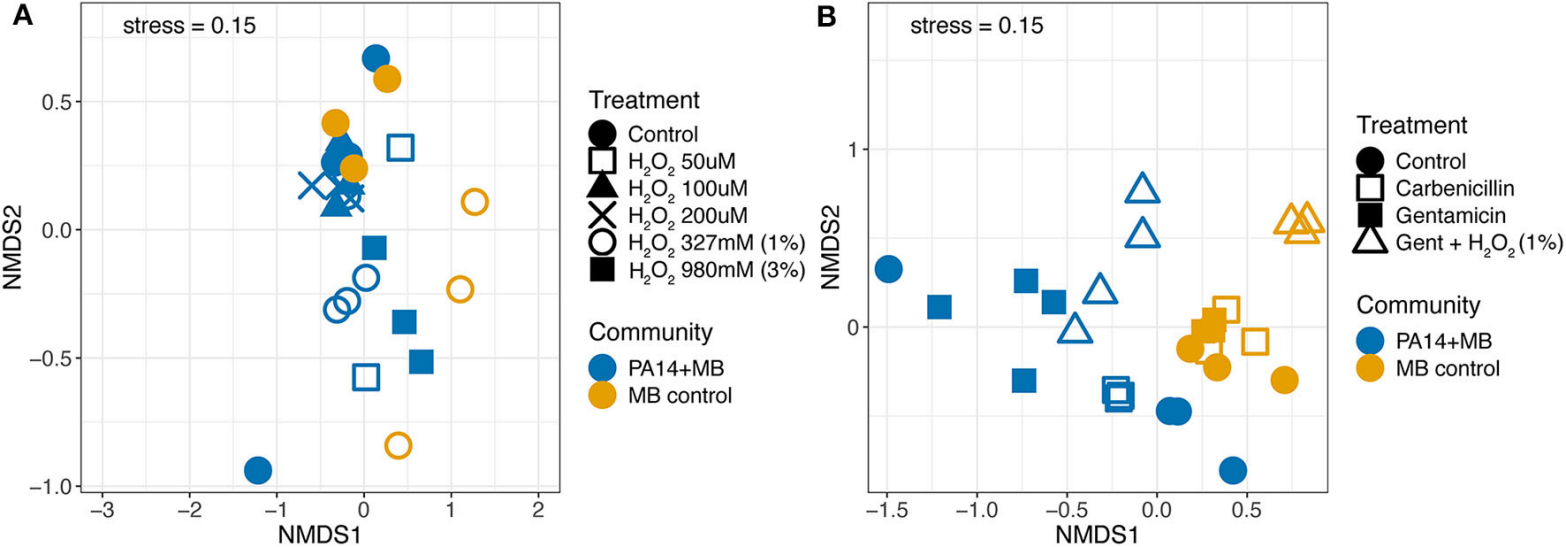
Non-metric multidimensional scaling (NMDS) plot of volatile signatures from the perfusion model from **(A)** hydrogen peroxide conditions and the control and **(B)** the antibiotic conditions and the control. The PA14+MB community represents the microbial community on a meat sample with both PA14 and MB(PA14) populations. The MB control community is a meat sample with only the meat microbial background. Each point represents an individual sample. *N* ≥ 3 biological replicates for all treatments.

Next, we used non-metric multidimensional scaling (NMDS) to visualize similarities in volatile signatures produced by the different treatment conditions. Figure 5A shows a comparison of volatile signatures from models treated with various concentrations of hydrogen peroxide. The control treatment for PA14+MB and MB control communities clustered together, with the exception of one outlier sample from PA14+MB on the bottom left. The MB control with 327 mM (1%) hydrogen peroxide was shifted away from the MB control treatment. PA14+MB with 327 mM (1%) and 980 mM (3%) hydrogen peroxide showed more distinct shifts away from the PA14+MB community control condition compared to PA14+MB community with lower concentrations of hydrogen peroxide (50–200 μM). One of the PA14+MB 50 μM hydrogen peroxide biological replicates, however, was more similar to PA14+MB 980 mM (3%) hydrogen peroxide than the control treatment.

Antibiotic treatments distinctly altered the abundances of volatile compounds. Here, the PA14+MB and MB control communities formed distinct clusters (Figure 5B). Within the MB control cluster, there was less movement with the addition of gentamicin or carbenicillin. MB control communities with the addition of gentamicin and hydrogen peroxide were shifted away from the main MB control cluster. Treatments for the PA14+MB community were more dispersed than the MB control. The PA14+MB community treated with antibiotics formed distinct clusters with biological replicates in close proximity to each other. PA14+MB with carbenicillin was more similar to the PA14+MB control than to gentamicin and gentamicin plus hydrogen peroxide. Similar to the NMDS with hydrogen peroxide and control treatments, PA14+MB control had one sample cluster closer to PA14+MB treated with gentamicin than to the control treatment samples. In summary, antibiotics were driving changes in the volatile composition, whereas hydrogen peroxide did not appear to have as large of an impact.

**Figure 6:**
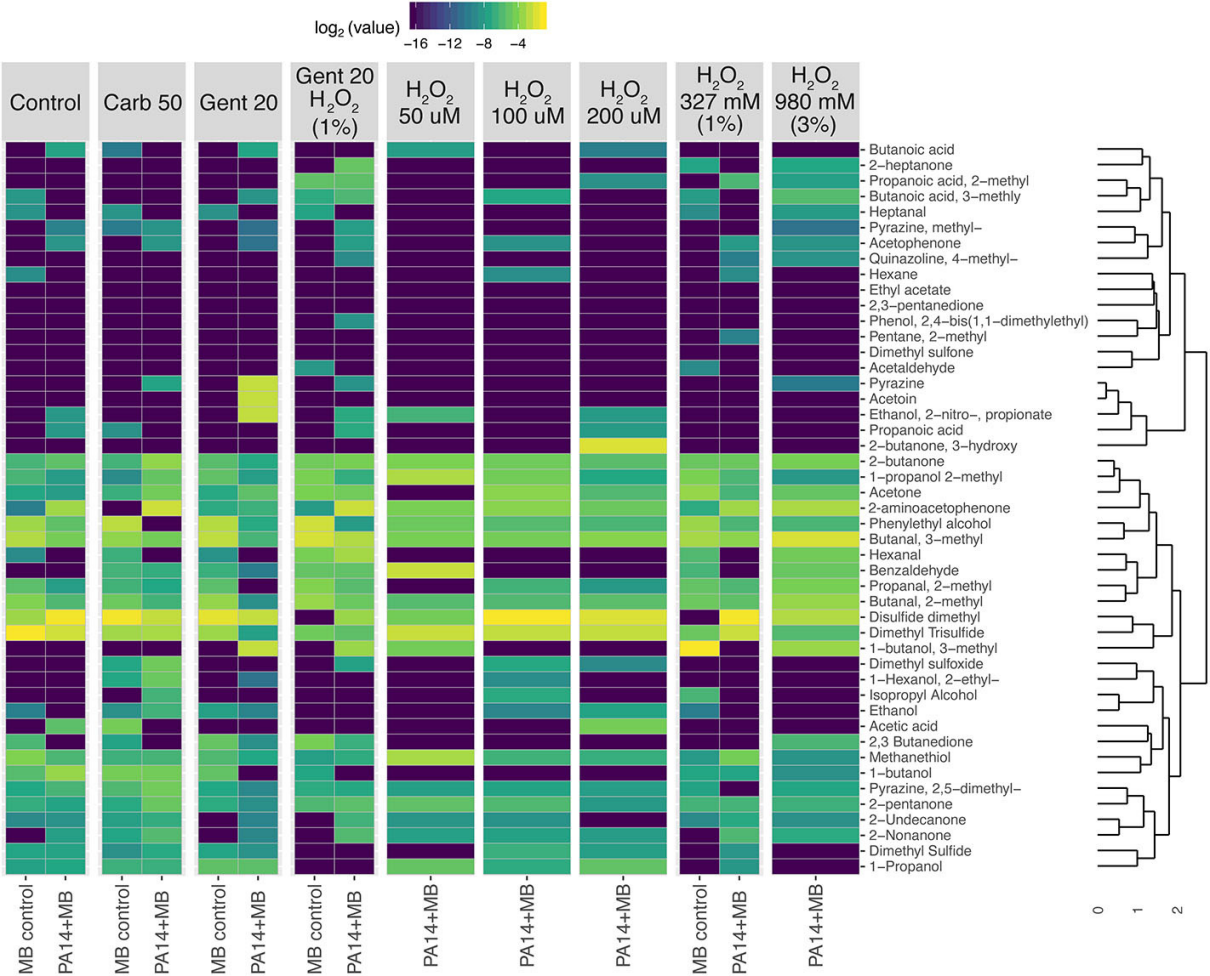
Heatmap showing clustering of relative abundances of volatile metabolites. PA14+MB represents the microbial community on a meat sample with both PA14 and MB(PA14) populations. The MB control community is a meat sample with only the meat microbial background. Metabolites were first normalized by total sample intensity. A Bray-Curtis distance matrix was generated for clustering with the unweighted pair group method with arithmetic mean (UPGMA) method.

**Figure 7:**
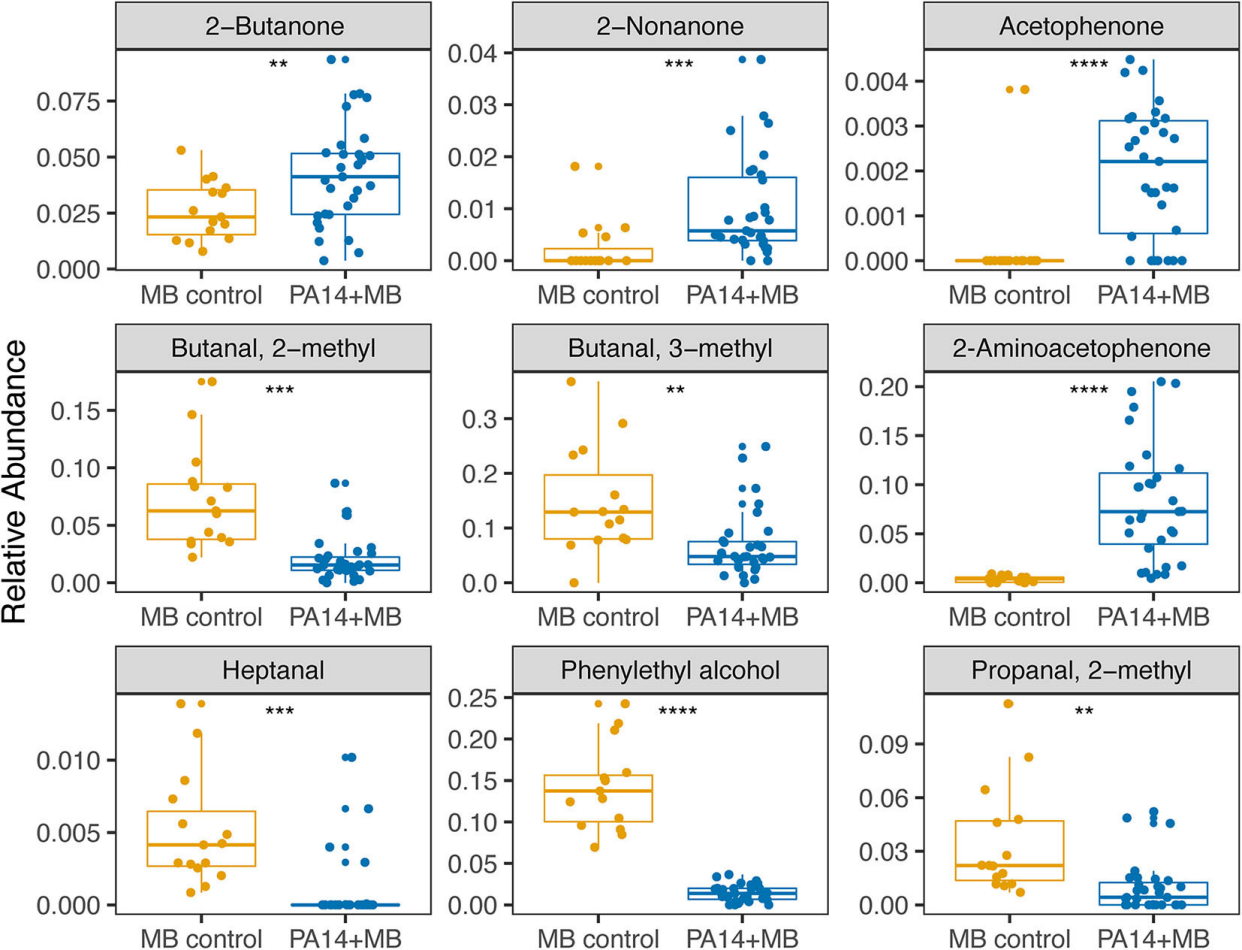
Relative abundances of metabolites significantly different between PA14+MB and MB control communities. PA14+MB represents the microbial community on a meat sample with both PA14 and MB(PA14) populations. The MB control community is a meat sample with only the meat microbial background. The relative abundances are values resulting from normalizing by total sample intensity. Each point represents a biological sample. All treatments are represented in this figure. Statistical testing was performed with *T*-tests. Significance is indicated by asterisks. **p < 0.01; ***p < 0.001; ****p < 0.0001.

Taking a closer look at the volatile molecules detected in each perfusion treatment condition, we saw that many highly abundant volatile metabolites were present across all conditions while some were more condition-specific (Figure 6). The classes of molecules we were able to detect included alcohols, aldehydes, aromatic compounds, carboxylic acids and esters, hydrocarbons, ketones, and sulfuric molecules. Acetophenone and 2-aminoacetophenone were mostly present only in PA14+MB community treatments and had significantly higher relative abundances in the PA14+MB community compared to the MB control community (*p*-value = 6 × 10^−5^ and *p*-value = 4 × 10^−6^, respectively; Figure 7). Additional metabolites that were significantly more abundant in PA14+MB than MB control were 2-nonanone and 2-butanone (*p*-value = 0.006 and *p*-value = 0.029, respectively; Figure 7). Across antibiotic and H_2_O_2_ treatments applied to the PA14+MB community, 2-aminoacetophenone and 2-butanone were significantly more abundant with carbenicillin compared to control (*p*.adj = 0.0066 and *p*.adj = 0.0041) and 2-butanone was significantly more abundant with gentamicin + hydrogen peroxide treatment (*p*.adj = 0.03; Supplementary Figure 3). As for the MB control community, butanal, 2-methyl; butanal, 3-methyl; heptanal; phenylethyl alcohol; and propanal,2-methyl were significantly more abundant in MB control than PA14+MB community (Figure 7). The MB control community had significant higher abundances of butanal, 3-methyl and propanal, 2-methyl in the gentamicin + hydrogen peroxide treatment compared to control (*p*.adj = 0.012 and *p*.adj = 0.042; Supplementary Figure 3). Thus, in addition to the global changes shown in Figure 4, we were also able to detect changes in individual metabolites across treatments, suggesting a combination of metabolites are important for identifying responses to antibiotics and hydrogen peroxide.

## Discussion

The purpose of our study was to develop and test a novel three-dimensional *in vitro* perfusion meat model in order to study the effects of antimicrobials on polymicrobial communities in the presence of pathogens. The main questions this research addressed were: (1) how does the microbial community growth shift in response to antibiotics and hydrogen peroxide, molecules commonly present in a chronic wound and (2) how does metabolism change in response to these compounds? We investigated *P. aeruginosa* because of its widespread relevance in chronic infections. We were also interested in the native microbial community on the meat to model an infection in the context of a polymicrobial community and include the potential colonization of wounds by a background commensal population. We found that we were able to culture *P. aeruginosa* in a perfused media model and that there were growth and metabolic consequences on the polymicrobial community in response to antibiotic and hydrogen peroxide treatments.

Our perfusion meat model is the first *in vitro* model to include the combined elements of tissue, flow, polymicrobial community growth, and volatile metabolomics to investigate bacterial community development *in vitro*. Past and current *in vivo* and *in vitro* models have greatly contributed to the understanding of wound healing physiology and biofilm structure, yet there are some important factors that we should take into consideration. A new review by Thaarup and Bjarnsholt (2020) highlights the pros and cons of the different model characteristics of current *in vitro* chronic wound models. Porcine skin is a widely used model for chronic wounds because it is more similar to human skin than rodent models (Montagna et al., 1964; Lindblad, 2008; Seaton et al., 2015). However, physiological differences between pig and human skin may confound results from pig skin models. For example, pig skin and hair follicles are less vascular and the sweat glands differ (Montagna et al., 1964).

Although wound infections that reach skeletal muscle are rare and understudied in *in vivo* models (Tzika et al., 2013; Bandyopadhaya et al., 2016a), our choice of meat provides a novel framework which enables us to study microbial metabolism in a biologically relevant context *in vitro*. Examples of infections that reach muscle, resulting from abscess formation, trauma, or surgery, are called infectious myositis (Bickels et al., 2002; Crum-Cianflone, 2008). The types of agents in infectious myositis are bacteria, fungi, parasitic, and viral (Bickels et al., 2002; Crum-Cianflone, 2008). There are currently no *in vitro* polymicrobial infection models in muscle tissue. Additionally, microbial biofilms are not studied widely in porcine skin models. The MB community in our model is a novel component as it likely comes from environmental contaminants and endogenous sources from the meat. One caveat of our study is the sequencing of the MB community from the static model, which identifies the microbes that grow on meat and the surrounding liquid. The microbes identified are likely to be the same as those that could grow on the surface of the meat in the perfusion model, but further isolate or metagenomic sequencing from the perfusion model will reveal characteristics of the background microbial community. Another concern is that as the meat comes from a commercial source, and we do not know what effect freezing (for storage) has on the microbiome or structure of meat and should be considered in future studies.

Perfusion is an important biological process that may contribute to wound healing in infections. During the inflammatory phase in wound healing, essential cells (e.g., antibodies, white blood cells, growth factors, enzymes, and nutrients) reach the wounded area by blood vessel dilation. Following dilation, the tissue can receive oxygen and nutrients through the proliferation phase in which the network of blood vessels is restored by growth factors (angiogenesis) (Landén et al., 2016). However, perfusion in chronic wounds is limited, resulting in low oxygenation and accumulation of harmful byproducts that delay healing (Woo et al., 2015). Having control over the perfusion rate and perfusion media in our model would be a step towardz recapitulating the physiological role blood vessels play in mediating chronic infections. In addition, the model is amenable to microscopy and has throughput and research regulatory advantages over *in vivo* models. When comparing perfusion and static models, we observed significantly higher growth of the MB control population in the control, antibiotic, and hydrogen peroxide treatments under the perfusion model (Figure 2). A potential explanation for differences in growth in the MB control community is oxygen and nutrient availability in the perfusion model compared to the static model. As the static model was submerged in media, there was less oxygen and fewer nutrients available to the microbes underneath the surface of the liquid.

The compounds perfused through the model included molecules relevant for treating chronic wound infections. Antibiotics and hydrogen peroxide are routinely used against bacterial infections, and hydrogen peroxide is also produced by the host during wound healing (Lorentzen et al., 1996; Loo et al., 2012; Roy et al., 2006; Ojha et al., 2008; Lee et al., 2008; Hammond et al., 2011). Very early after injury, wounds with impaired healing contain increased levels of reactive oxygen and nitrogen species (Dhall et al., 2014). Low levels of reactive oxygen species (ROS) act as essential mediators of intracellular signaling and regulate numerous signal transduction and gene expression processes (Roy et al., 2006). ROS at low concentrations lead to defense against invading pathogens and proper healing. In contrast, high levels of ROS clearly have the potential to complicate regeneration and remodeling of nascent tissue. ROS can cause damage by reacting with nucleic acids, protein, and lipids and delay healing (Sen and Roy, 2008; Dhall et al., 2014). In our perfusion model, we used physiological μM and therapeutic mM concentrations of hydrogen peroxide and observed that *P. aeruginosa* cell densities reached around 10^9^-10^10^ CFU/ml. These densities were not significantly lower than the MB control under these conditions, with the exception of PA14 in gentamicin. There was also a decrease in total ion intensity as concentrations of hydrogen peroxide increased (Figure 4), indicating a change in microbial metabolism in response to hydrogen peroxide exposure. The suppression of MB growth in the presence of both the lab-adapted PA14 and PaFLR01 clinical isolate of *P. aeruginosa*, and when exposed to antimicrobial compounds, suggests an interaction between the pathogen and MB populations that is triggered by exposure to the chemotherapeutics. Differences in MB growth inhibition in the presence of either PA14 or PaFLR01 depending on exposure to carbenicillin or gentamicin (Figure 3) may be due to differences in each strain's response to each antibiotic. PaFLR01 is sensitive to carbenicillin and gentamicin at 20 μg/ml whereas PA14 is resistant to carbenicillin at 20 μg/ml and sensitive to gentamicin at 20 μg/ml (Supplementary Figure 4).

Metabolomic approaches have been extensively used to predict biomarkers of disease and bacterial colonization. The use of headspace metabolomics to capture volatile molecules is a powerful approach for capturing molecules that may act as important signals in a biological context and also as detectable biomarkers of the bacterial species and metabolic conditions. Common volatile compounds associated with *P. aeruginosa* that were detected in our study include acetophenone, 2-aminoacetophenone, dimethyl sulfide, dimethyl trisulfide, 2-butanone, 2-nonanone, and others (Labows et al., 1980; Shestivska et al., 2012; Bean et al., 2016; Ashrafi et al., 2017). However, only a subset of the volatile molecules associated with *P. aeruginosa* (acetophenone, 2-aminoacetophenone, 2-nonanone, and 2-butanone) were significantly higher in the PA14+MB community compared to the MB control community (Figure 7) and 2-aminoacetophenone and 2-butanone were significantly different across treatments (Supplementary Figure 1). 2-aminoacetophenone has been widely associated with identification of *P. aeruginosa* (Cox and Parker, 1979; Scott-Thomas et al., 2010; Pabary et al., 2016), with quorum sensing (Kesarwani et al., 2011; Kviatkovski et al., 2015), and with oxidative stress (Bandyopadhaya et al., 2016a). In a murine skeletal muscle model, 2-aminoacetophenone produced by *P. aeruginosa* promotes mitochondrial dysfunction and reduced energy production (Tzika et al., 2013) by inducing oxidative stress and apoptosis signaling pathways (Bandyopadhaya et al., 2016a). 2-aminoacetopheone has also been shown to alter the host epigenome, enabling higher bacterial burden tolerance (Bandyopadhaya et al., 2016b) and promotes chronic infection (Kesarwani et al., 2011). Given the important role 2-aminoacetophenone plays in *P. aeruginosa* physiology, 2-aminoacetophenone could be an important biomarker for monitoring antibiotic efficacy in *P. aeruginosa*. The conditions that lead to the production of specific microbially derived metabolites associated with infection provide insight into wound physiology and outcome.

From a broader perspective of the volatile metabolome across all treatments, antibiotics had a greater influence on the volatile signature than hydrogen peroxide (Figure 5). The addition of carbenicillin, gentamicin, and gentamicin + hydrogen peroxide decreased the total volatile ion intensity detected in the PA14+MB community (Figure 4), while growth of the PA14 population was significantly higher than the MB(PA14) population for the carbenicillin treatment (Figure 3). This implies there was a diminished microbial metabolic response to the treatments for the MB(PA14) population, suggesting that the bulk of the volatile signal may be coming from the meat microbial community. *P. aeruginosa* may be thriving in the stressful environment generated by exposure to the chemotherapeutics, and it is outcompeting its corresponding MB community, especially in the case with carbenicillin, hydrogen peroxide (1–980 mM), and gentamicin + hydrogen peroxide for PA14, and gentamicin and 327 mM (1%) hydrogen peroxide for PaFLR01 (Figure 3, Supplementary Figure 1). Some possibilities for *P. aeruginosa* outcompeting the MB include cross-feeding changing the stress tolerance of *P. aeruginosa*, the MB may be more susceptible to antibiotics and hydrogen peroxide, or *P. aeruginosa* may be more adapted to more stressful environments than the MB. Lower concentrations (50–200 μM) of hydrogen peroxide, in the range of physiological concentrations, had higher ion intensities than the untreated controls, suggesting increased microbial metabolism. In addition, there were no significant differences in CFU/ml between PA14 and MB(PA14) populations for hydrogen peroxide conditions from 50 to 500 μM (Supplementary Figure 1). Together with the use of multiple volatile molecules to serve as a fingerprint for *P. aeruginosa* metabolism, understanding which organisms contribute to the bulk of the volatile signal is also informative for identifying changes community physiology.

The perfusion meat model is promising for the study of clinical wound isolates in more physiologically relevant conditions. In chronic infections treated with antibiotics, an individual pathogen such as *P. aeruginosa* comes to dominate over time. In the present model, lab-adapted and clinical *P. aeruginosa* strains reached higher cell densities than their respective MB populations when treated with carbenicillin, gentamicin, or hydrogen peroxide. The model may help us understand why common opportunistic pathogens thrive in infections despite treatment with antibiotics and reactive oxygen species. Furthermore, our goal of identifying microbial metabolism in response to different treatments is being realized with the use of volatile headspace analysis and metabolomics. Moreover, an important aspect of an *in vitro* model is accessibility. While our model surely does not recapitulate *in vivo* behavior in all cases, its ultimate utility is a function of both its ability to do so in some cases and its accessibility to other researchers, which we believe is an additional advantage of our model over others. For future studies aimed at identifying biomarkers for wound chronicity, it is important to utilize metabolomics to identify changes in metabolism for more targeted therapies, and to use metagenomics for a species-specific investigation of how the microbial community affects how pathogens invade wounds. There may be antagonistic effects of the native MB microbiota in the meat against *P. aeruginosa*; isolate sequencing to identify the species level identity would allow us to further investigate the relationship between specific bacteria. The use of biologically relevant models, such as our perfusion meat model, would also advance the field for understanding polymicrobial interactions and metabolisms within a chronic infection.

## Data Availability Statement

16S rRNA amplicon sequence data were deposited on the National Center for Biotechnology Information (NCBI) sequence read archive under the BioProject accession number PRJNA640161 and can be found here: https://www.ncbi.nlm.nih.gov/bioproject/PRJNA640161/. Metabolomics data and R scripts for statistical analysis are published on GitHub at https://github.com/joannlp/perfusion.

## Author Contributions

JP, SR, KW, and AH contributed to the design of the research. KW and AH supervised the project. JP and SR carried out the perfusion experiments. JP carried out the static experiments and the volatilome analysis. MK performed the headspace extractions and analysis of the data generated from the GC-MS. MG performed the 16S rRNA sequencing and analysis. JP generated all of the figures. JP, SR, AH, and KW contributed to the interpretation of results. JP and SR took the lead in writing the manuscript. All authors provided feedback and helped shape the research, analysis, and manuscript.

## Conflict of Interest

The authors declare that the research was conducted in the absence of any commercial or financial relationships that could be construed as a potential conflict of interest.
