# Supplementary figures and images for: Thriving Under Stress: Pseudomonas aeruginosa Outcompetes the Background Polymicrobial Community Under Treatment Conditions in a Novel Chronic Wound Model
Source: Front Cell Infect Microbiol. 2020 Oct 6;10:569685. doi: 10.3389/fcimb.2020.569685 (PMC7573134; doi:10.3389/fcimb.2020.569685)

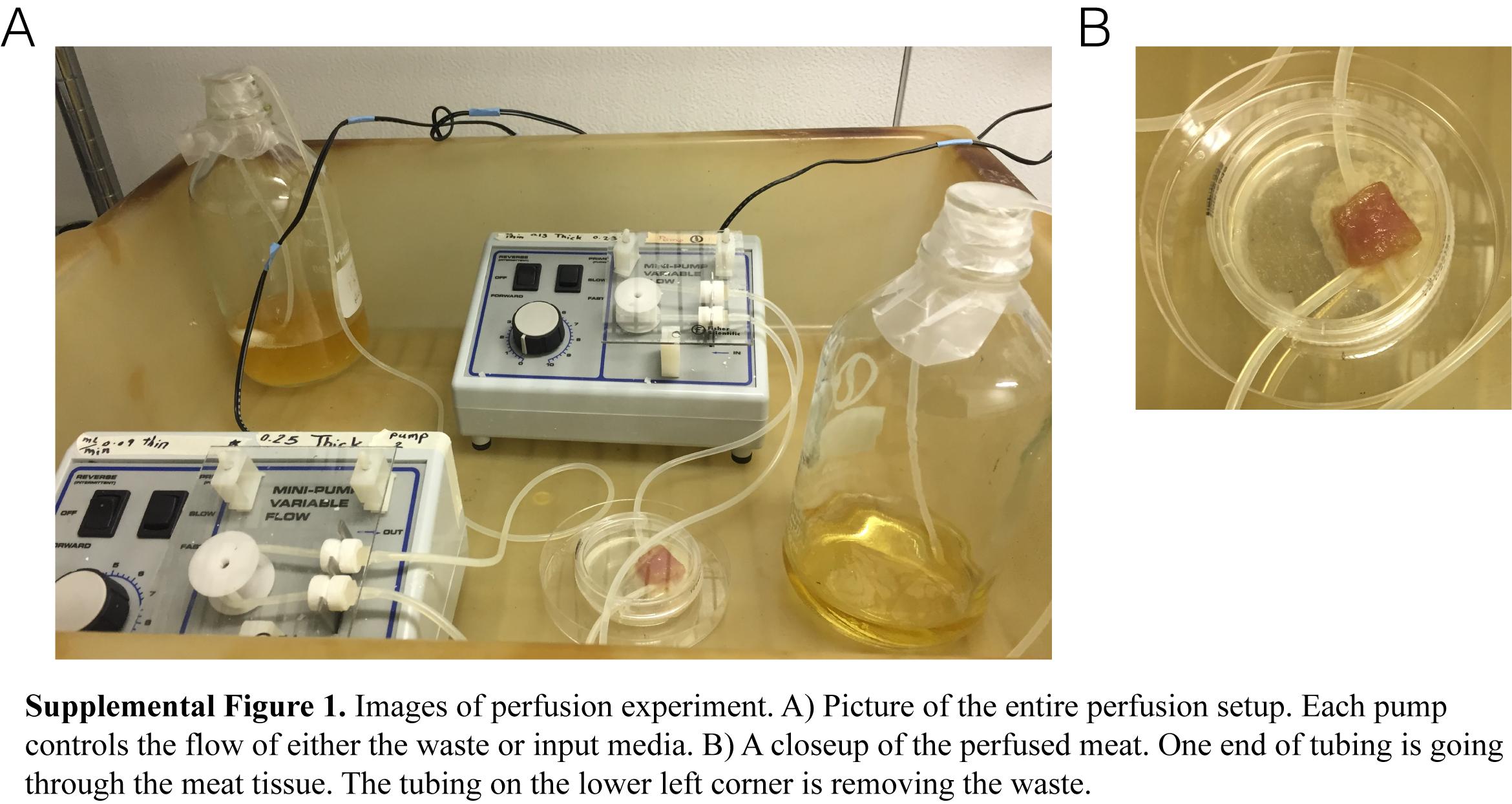

Supplement: Supplementary file 2 [file Image_1.TIF]

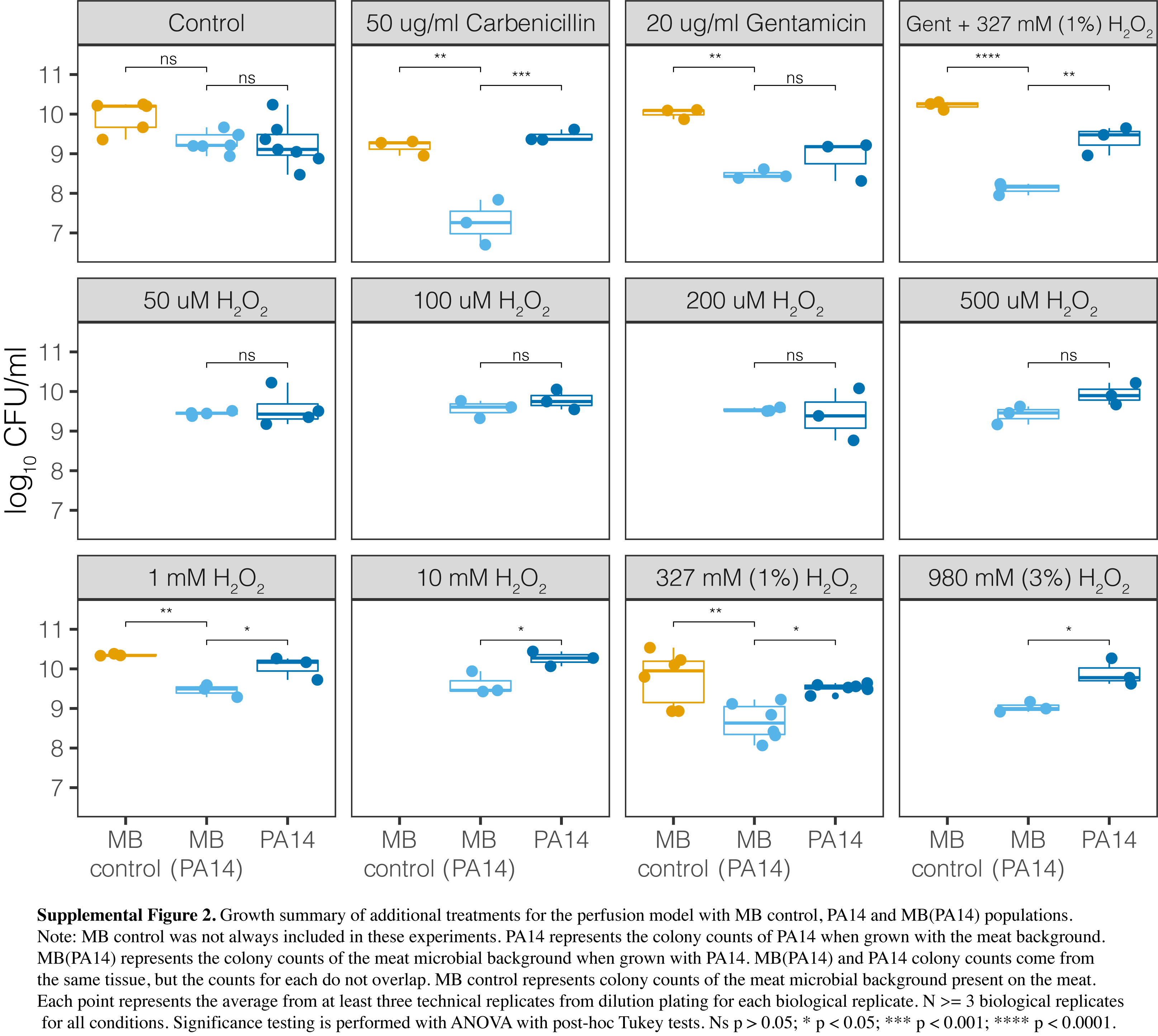

Supplement: Supplementary file 3 [file Image_2.TIF]

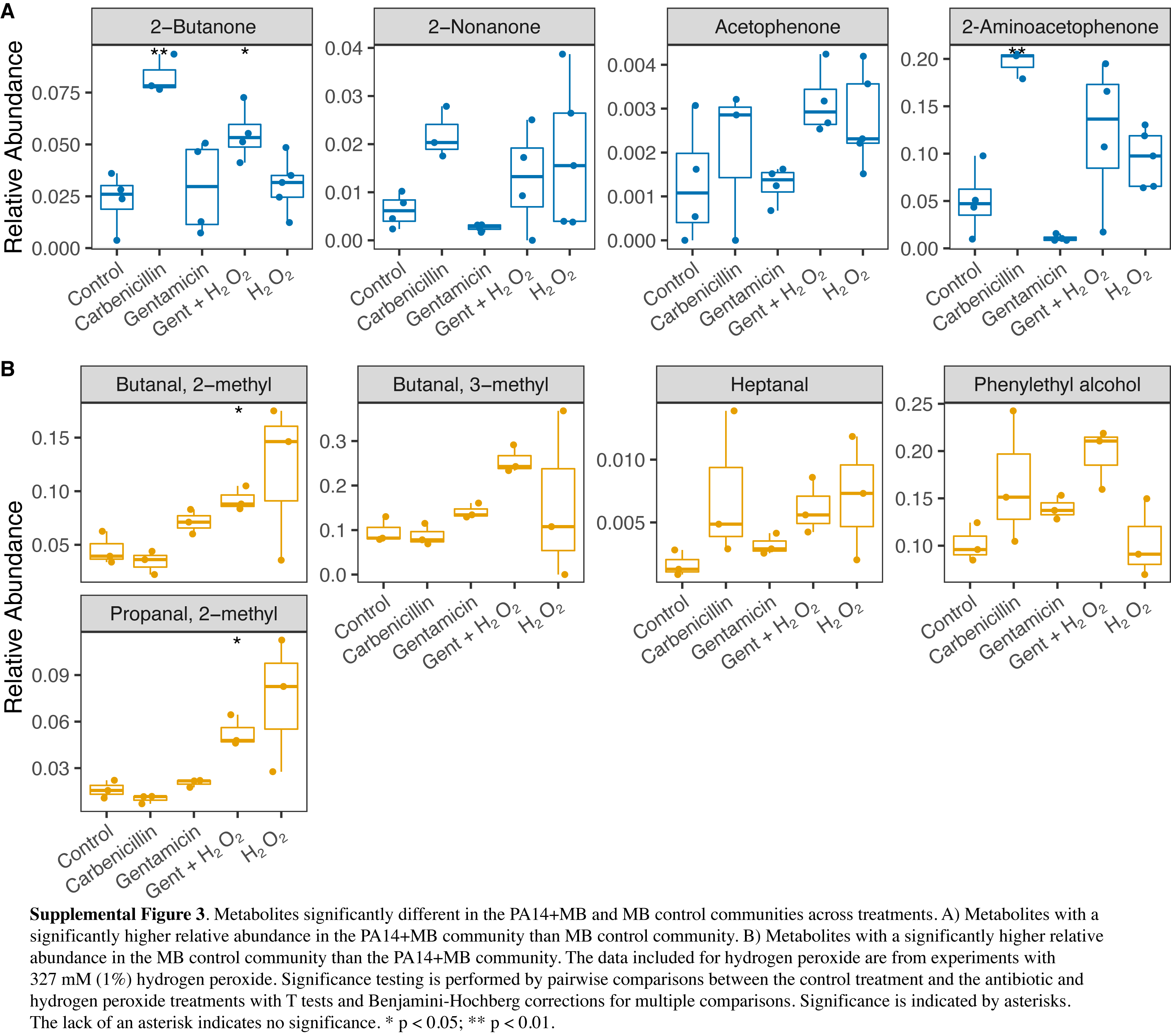

Supplement: Supplementary file 4 [file Image_3.TIF]

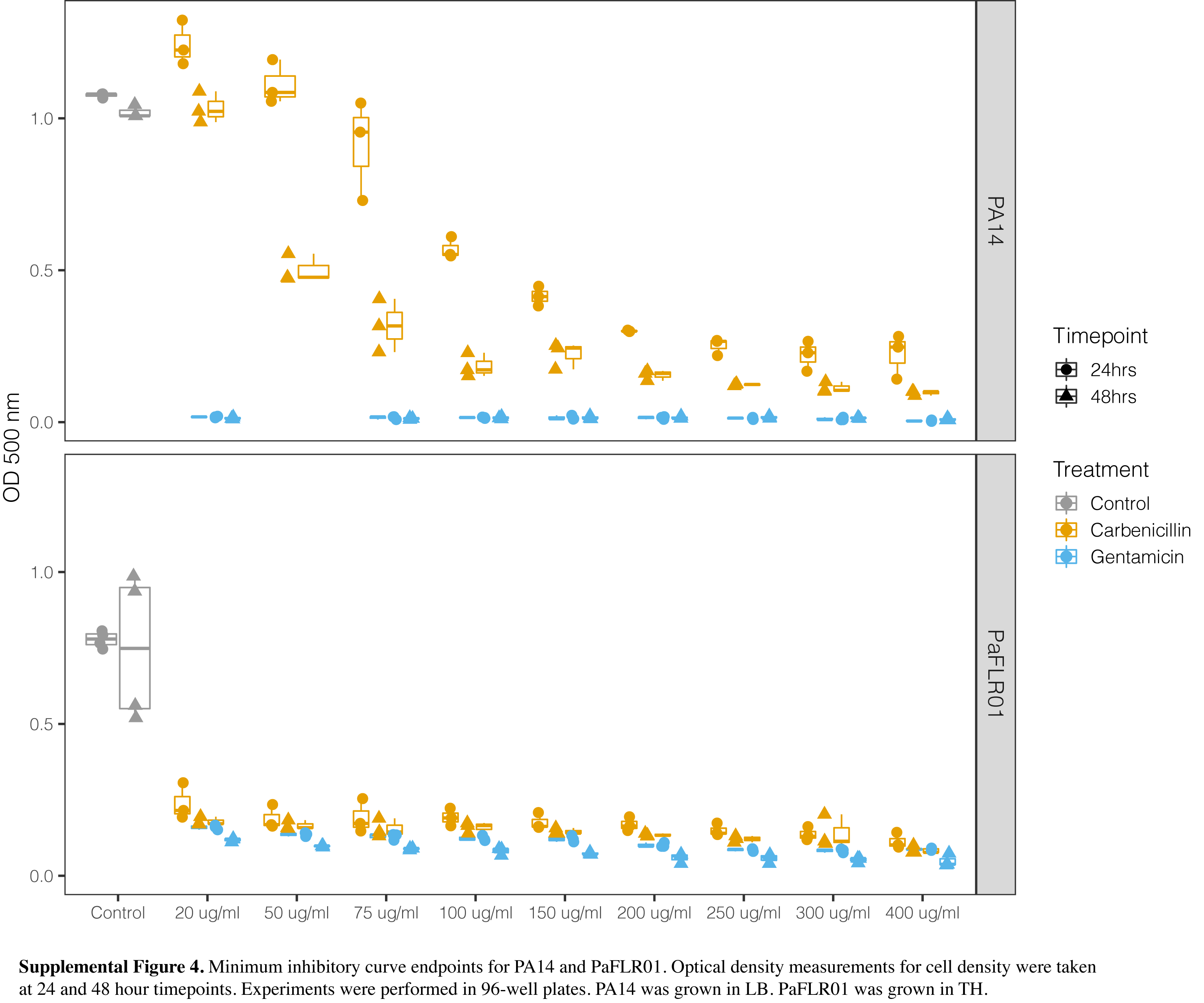

Supplement: Supplementary file 5 [file Image_4.TIF]
